# Valorisation of *Posidonia oceanica* Sea Balls (Egagropili) as a Potential Source of Reinforcement Agents in Protein-Based Biocomposites

**DOI:** 10.3390/polym12122788

**Published:** 2020-11-25

**Authors:** Seyedeh Fatemeh Mirpoor, Concetta Valeria L. Giosafatto, Prospero Di Pierro, Rocco Di Girolamo, Carlos Regalado-González, Raffaele Porta

**Affiliations:** 1 Department of Chemical Sciences, University of Naples “Federico II”, Montesantangelo Campus, via Cintia 4, 80126 Naples, Italy; seyedehfatemeh.mirpoor@unina.it (S.F.M.); prospero.dipierro@unina.it (P.D.P.); rocco.digirolamo@unina.it (R.D.G.); 2 Departamento de Investigacion y Posgrado en Alimentos, Facultad de Quimica, Universidad Autonoma de Queretaro, Queretaro 76010, Mexico; carlosr@uaq.mx

**Keywords:** *Posidonia oceanica*, egagropili, nanocrystalline cellulose, lignin, protein-based biocomposites

## Abstract

Nanocrystalline cellulose (NC) and a lignin-containing fraction (LF) were obtained from egagropili, the so called sea balls produced from rhizome and stem fragments of *Posidonia oceanica* that accumulate in large amounts along the coastal beaches in the form of tightly packed and dry materials of various dimensions. Both egagropili fractions have been shown to be able to improve the physicochemical properties of biodegradable films prepared from protein concentrates derived from hemp oilseed cakes. These materials, manufactured with a biodegradable industrial by-product and grafted with equally biodegradable waste-derived additives, exhibited an acceptable resistance with a still high flexibility, as well as they showed an effective barrier activity against water vapor and gases (O_2_ and CO_2_). Furthermore, both NC and LF decreased film moisture content, swelling ability and solubility, thus indicating that both additives were able to improve water resistance of the hydrocolloid films. The exploitation of egagropili, actually considered only an undesirable waste to be disposed, as a renewable source of reinforcing agents to blend with different kinds of polymers is suggested.

## 1. Introduction

Plastics pollution has become a global threat, mostly to marine ecosystems [1]. Therefore, replacing oil-derived plastics with biodegradable materials is required by a more sustainable life pursuit and, in this respect, polymer matrices derived from bio-renewable polysaccharides and proteins are of highlighted interest. Numerous researches have been carried out in the last twenty years on using polysaccharide fibers derived from agriculture wastes or industrial by-products to develop biodegradable/edible films with improved performance [2].

**Figure 1:**
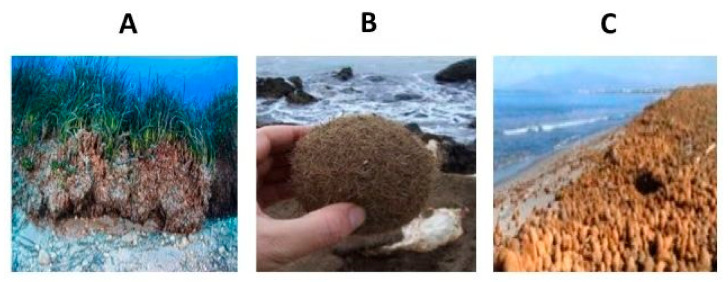
*Posidonia oceanica* rhizomes and stems (**A**) from which ball shaped dry materials called egagropili (**B**) origin and accumulate along the costal beach (**C**).

Biocomposites containing plant- or wood-based fibers have been exploited thus far in an increasing range of items to reinforce plastics, having several advantages over synthetic additives. Plant cell walls consist essentially of rigid cellulosic microfibrils embedded in a soft matrix of hemicellulose and lignin, and cellulose is the major component in lignocellulosic biomass which is localized in the cell wall at around 35–50% [3]. Lignin represents a fairly stable polymer network that acts as a glue to hold the other matrix components (cellulose/hemicellulose) together [4]. Although most of the starting raw materials used are land vegetable sources, aquatic biomass, such as marine plants, may represent a possible effective alternative because the chemical composition of their fibers is similar to that of other lignocellulosic materials. In particular, *Posidonia oceanica* (PO) is one of the most abundant Mediterranean endemic species, covering 60% of the seabed from 0 to 40 m depth [5] and about fifty thousand km^2^ of coastal sandy areas [6]. One of the largest PO patches in the Mediterranean Sea contains forty undersea meadows stretching over two thousand miles. PO has been demonstrated to be an optimum source for the extraction of lignocellulosic fractions with promising properties for the development of packaging materials or to be used as fillers to enhance the properties of other biopolymers [7,8,9,10]. Cellulose extracted from PO, for example, has been proposed as a source of both carboxymethylcellulose [11,12] and sodium cellulose carboxymethylate [13] used as absorption materials. However, whereas stranded PO leaf residues play an important ecological role in protecting of coasts from erosion, other PO fragments accumulate in large amounts along the coastal beaches in the form of tightly packed and ball-shaped (oval or spherical) dry materials of different dimensions, called “egagropili”. In fact, what remains of PO fiber-like leaves that are at the base of rhizomes and stems cluster together very closely and generate these characteristic free-floating and brown colored balls. The sea balls, also called sea rissoles or sea potatoes, are similar in texture to rough felt and tend to form by long-wavelength surface waves that run up on flat beaches. Wind and the tide push them together and, once completely dry, they accumulate in huge numbers along the sandy shores (Figure 1). Since egagropili represent a problem, first of all for their negative visual impact [14], the municipalities are forced to remove and dispose them in landfills as municipal wastes with non- negligible costs.

Therefore, the search for turning egagropili into a resource that provides economic benefits and positive feedback from an environmental point of view is strongly advisable because egagropili do not significantly differ from other lignocellulosic materials [12]. Their small salt content (0.5–2%) allows egagropili to resist decomposition and, being virtually non-flammable, they are currently being studied for insulation use since they markedly reduce heat loss. In addition the dry residue of egagropili is made of holocellulose, lignin and a small amount of ash. Holocellulose is the total carbohydrate component and has been calculated to be 61.8% where cellulose contributes 40%, making this fraction particularly suitable as renewable cellulose source [15]. Among the numerous published studies on the use of lignocellulosic fibers as additives of biodegradable materials [2], Seggiani et al. [16] developed polyhydroxyalkanoate-based bio-composites specifically grafted with PO fibers for potential applications in marine environment, such as natural engineering interventions.

In this paper both cellulose and lignin containing fractions obtained from egagropili fibers were investigated to assess their properties for a possible use as reinforcement additives of protein-based biodegradable/edible films, potentially able to replace oil-based plastics in packaging systems.

## 2. Materials and Methods

### 2.1. Materials

Hemp oilseed cakes, a generous gift of prof. Daniele Naviglio, were purchased from Consorzio Goji Italia (Andria, Italy). Egagropili sea balls were collected in the sardinian Poetto beach (Cagliari, Italy) and stored at 4 °C until used. Sodium hydroxide, hydrochloric acid, sodium chlorite, acetic acid glacial, potassium hydroxide, sulfuric acid and glycerol (GLY) were purchased from Sigma Chemical Co (St. Louis, MO, USA). All other chemicals were of analytical grade.

### 2.2. Egagropili Lignin Containing Fraction and Nanocrystalline Cellulose

Egagropili balls were reshaped to rhizome fibers by hands, then washed and rinsed vigorously in distilled water in order to remove sand, salts and other soil contaminants, and finally dried in an oven at 80 °C for 24 h. The dried egagropili fibers were grinded in a rotary mill (Grindomix GM200, Retsch GmbH, Haan, Germany) at a speed of 1000 rpm for 3 min to a 60-mesh sieve size. Cellulose extraction was carried out as reported by Ilyas et al. [17] with some modifications. In the first step, 20 g of grinded egagropili fibers were dewaxed by means of Soxhlet apparatus, with 440 mL of toluene/ethanol (2:1 *v/v*) during 24 h and oven dried overnight at 105 °C. Afterwards, delignification process has been carried out by dispersing at 70 °C for 2 h the dewaxed egagropili powder in 600 mL of 1.7% sodium chlorite solution brought at pH 3.5 by acetic acid. This process was repeated 3 times consecutively until the color changed from brown to white/yellowish. The resulted bleached fibers, known as holocellulose, were filtered by using filter paper and washed with distilled water until the filtrate became neutral. The first filtrate obtained in the delignification step was referred as the lignin containing fraction (LF). A quantitative analysis, carried out by calculating the dry weight of LF, indicated that 152 mg of LF were obtained from 1 g of grinded egagropili powder. To remove hemicellulose and residual pectin, the obtained holocellulose was treated with 5% potassium hydroxide for 24 h at room temperature followed by an exposure for 2 h at 90 °C. Finally, the obtained cellulose fraction was filtered and washed several times until pH neutralization, and finally dried at 55 °C for 18 h. 

**Figure 2:**
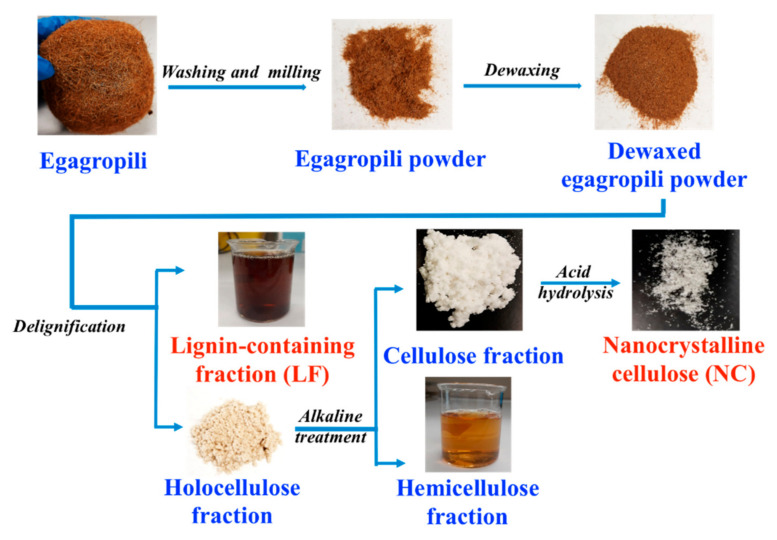
Scheme of the extraction procedure of nanocrystalline cellulose and lignin containing fraction from egagropili.

Nanocrystalline cellulose (NC) was obtained by sulfuric acid hydrolysis of the egagropili cellulose fraction according to the procedure described by Sanyang et al. [18]. Acid hydrolysis started by adding 1 g of cellulose fraction into 10 mL of sulfuric acid solution (65%, *w/w*) and by stirring the reaction mixture at 45 °C for 45 min in order to totally hydrolyze the amorphous regions of cellulose. Hydrolysis reaction was stopped by diluting ten-times the suspension by cold distilled water, followed by repeated washing of the pellet obtained by centrifugation at 10,000 rpm for 10 min, until the neutral pH was reached. The resulting precipitate containing NC was finally freeze-dried and, then, the obtained powder was dispersed in distilled water and subjected to ultrasonication for 10 min at 400 W to stabilize NC dispersion by eliminating its excessive aggregation. The quantitative gravimetric determination indicated that 225 mg of NC were obtained from 1 g of grinded egagropili powder. The entire extraction process, summarized in Figure 2, was monitored by Fourier-transform infrared spectroscopy analysis using a model ALPHA spectrometer (Bruker, Leipzig, Germany) equipped with an attenuated total reflectance accessory.

### 2.3. Preparation of Protein-Based Composite Films

Hemp proteins (HPs) were extracted from the industrial hemp seed oilcake, a byproduct obtained after the extraction of the hemp seed oil. Defatted hemp seed flour was treated under alkaline conditions (pH 11), and the supernatant obtained after centrifugation was precipitated at acidic pH (5.4). The obtained pellet (HP concentrate) was finally dried and used to obtain hydrocolloid films. The protein content of the derived final HP concentrate was determined by the Kjeldahl’s method [19], using a nitrogen conversion factor of 6.25. HP stock solution (2% *v/w*) were prepared by dissolving the protein concentrate in distilled water and by adding 1 N NaOH, under constant stirring at room temperature, until the pH of the solution was brought at pH 9 or 12. Film forming solutions (FFSs) (25 mL) were prepared at the two different pH values by using 400 mg of HPs in the presence of 50% GLY, used as plasticizer, and different amounts of either NC (at pH 9) or the extensively dialyzed LF (at pH 12) extracted from egagropili. All kinds of FFSs were cast onto 8 cm diameter polycarbonate Petri dishes and allowed to dry in an environmental chamber at 25 °C and 45% relative humidity (RH) for 24 h. The dried films were peeled, intact, from the casting surface and analyzed after their conditioning at 50% RH and 25 °C by placing them in a desiccator over a saturated solution of Mg(NO_3_)_2_·6H_2_O for 24 h.

### 2.4. Zeta Potential and Particle Size Measurements

The effect of both NC and LF extracted from egagropili on zeta potential and particle size of the HP containing FFSs at pH 9 or 12, respectively, was studied. To this aim each FFS (1.0 mL), containing 50% GLY and 16 mg HPs, was tested in the absence or presence of different amounts of either NC or LF. Zeta potential and particle size values were measured with a Zetasizer Nano-ZSP (Malvern^®^, Worcestershire, UK) in the wavelength of 633 nm using a helium-neon laser of 4 mW output power. Zeta potential was calculated by the instrument software programmer trough the electrophoretic mobility at a voltage of 200 mV using the Henry equation. Zeta potential and particle size measurements were performed at each pH in triplicate and all the results were reported as mean ± standard deviation.

### 2.5. Film Mechanical Properties

Film tensile strength (TS), elongation at break (EB) and Young’s modulus (YM) were measured by an Instron universal testing instrument (model no. 5543A, Instron Engineering Corp., Norwood, MA, USA). Each prepared film was cut into strips with a width of 10 mm and a length of 60 mm and at least three samples of each film were then tested, by using 1 kN load and 5 mm/min speed, as previously described [20]. Film thickness was determined randomly in five different locations by using a micrometer (IP65 Alpa exacto, Alpa metrology, Pontoglio (BS), Italy) with a precision of 0.001 mm.

### 2.6. Film Moisture Content, Swelling Ratio and Solubility

Film moisture content was analyzed on 3 × 3 cm^2^ samples according to the method described by Zahedi et al. [21]. Films were placed in the aluminum plates and dried at 105 °C in an oven for 24 h. Moisture content of the films was evaluated by calculating the difference between their weight before and after drying using the following equation:

Moisture content = [(*W*_i_ − *W*_d_)/*W*_i_] × 100 (1)

where, *W*_i_ and *W*_d_ represent the film weights before and after drying, respectively.

Film swelling ratio was determined by a gravimetric method [22]. Each film (*W*_i_) was immersed in 30 mL of distilled water at 25 °C for 1 h and, after drying of its surface by an absorbent paper, each film was finally weighed again (*W*_s_). The swelling ratio was calculated using the following equation:

Swelling ratio = [(*W*_s_ − *W*_i_)/*W*_i_] × 100 (2)

In order to determine film water solubility, the dried film was weighed (*W*_i_), immersed in 30 mL distilled water and shaken for 24 h at 25 °C. The undissolved film residues were finally dried in the oven at 105 °C to calculate the film dry weights (*W*_f_). Film solubility was determined as the percentage of total weight by using the following equation [23]:

Solubility (%) = [(*W*_i_ − *W*_f_)/*W*_i_] × 100 (3)

All the experiments were repeated at least three times.

### 2.7. Film Water Vapor, O_2_ and CO_2_ Permeability

Film water vapor (WV) and gas permeability was investigated, after film conditioning for 24 h at 50% RH, as previously described [24,25,26] by using a Total Perm apparatus (ExtraSolution s.r.l., Pisa, Italy) and aluminum masks to reduce the film test area to 5 cm^2^. All the experiments were repeated at least three times.

### 2.8. Scanning Electron Microscopy (SEM)

Microstructures of both grinded egagropili fibers and nanocrystalline cellulose were observed by using a Philips-FEI SEM (Nova NanoSem 450-FEI-Thermo Fisher, Scientific, Waltham, MA, USA). The dried samples were coated with thin layers of gold and platinum using a sputter coater at a current of 20 mA for 90 s and then the images were taken at an accelerating voltage of 3 kV, (4.4–5.2) mm working. Further surface and cross-section images were also obtained by analyzing hemp protein films derived from FFSs prepared in the absence or presence of either 6% NC or LF. For cross-section imaging, the samples were previously frozen using liquid nitrogen and then cryofractured.

### 2.9. Statistical Analysis

SPSS19 (Version 19, SPSS Inc., Chicago, IL, USA) software was used for all statistical analyses. One-way analysis of variance (ANOVA) and Duncan’s multiple range tests (*p* < 0.05) were used to determine the significant difference among the samples.

## 3. Results and Discussion

### 3.1. Egagropili as Potential Source of Lignin and Nanocrystalline Cellulose

Different fractions of egagropili powder were obtained by preliminary washing, de-waxing, heating and grinding of the starting material and, then, by delignification with sodium chlorite and separation of cellulose from hemicellulose and pectin by potassium hydroxide treatment. Finally, NC was obtained by sulfuric acid hydrolysis of the extracted cellulose to eliminate the amorphous regions of the polysaccharide. It is well known that NC is widely utilized as a reinforcement agent in polymer matrices for its peculiar properties such as a high crystallinity and aspect ratio, large specific surface area, as well as abundance of surface hydroxyl groups able to form hydrogen bonds [27]. Therefore, the aim of the present study was to investigate the possibility to exploit egagropili, until now considered by the local authorities as an undesired waste to be disposed, as a renewable resource to produce reinforced protein-based bioplastics, mainly thanks to their cellulose content. In addition, also the obtained egagropili LF was tested as possible reinforcement of the prepared protein-based films. Lignin is, in fact, the most recalcitrant component in lignocellulosic fibers, being extremely resistant to enzymes and chemical impacts [28] due to its polyphenolic structure which consists of three different phenylpropane monomeric units containing zero, one, and two methoxyl groups, respectively [29,30,31].

### 3.2. Surface Microstructure of Egagropili Fibers and the Derived Nanocrystalline Cellulose

**Figure 3:**
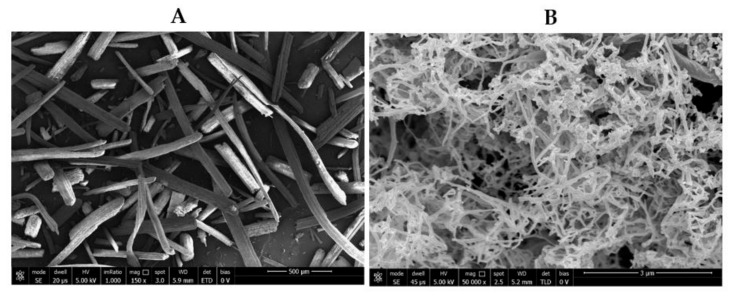
SEM images of both grinded egagropili fibers (**A**) and egagropili nanocrystalline cellulose (**B**).

The SEM image of surface microstructure of grinded egagropili fibers indicated that egagropili powder has a lignocellulosic fibrous structure and the determined fiber length was in the 1–2 mm range (Figure 3A). On the other hand, NC chains obtained by acidic hydrolysis of the cellulose fraction extracted from egagropili exhibited a rod-like structure of 65–90 nm in diameter with smooth surfaces. In addition, the NC chains appear not to be separated from each other leading to a nebulous morphology (Figure 3B). The observed agglomeration of this material was probably due, as previously reported [32], to the hydrophilic characteristics of cellulose.

### 3.3. Film Forming Solution Zeta Potential and Particle Size Measurements

FFSs containing HPs and different concentrations of NC (2, 4 and 6% with respect to HPs) were prepared at pH 9 and 1 mL of each FFS was analyzed to determine their zeta potential value and the particle size. Table 1 shows that the FFSs prepared in the presence of different amounts of NC exhibited zeta potential values very similar to that measured with the FFS containing only HPs, thus indicating that all the prepared FFSs were relatively stable [33,34]. Conversely, the mean value of the particle size significantly increased with the increase of NC concentration. This result could be probably due to the hydrogen bond formation between HPs and NC with the consequent formation of particles of higher dimensions. The larger standard deviation detected in the values obtained increasing NC concentration might depend on the variability of the dimension of the particles formed.

Furthermore, Table 1 also shows that the value of zeta potential of HP-based FFSs prepared at pH 12 in the presence of different concentrations of LF (3%, 6% and 9% with respect to HPs) was almost stable around −28 mV and that, in this case, also the mean particle size was not significantly influenced by the presence of different amounts of LF added to the FFS. It is worthy to note that the polydispersity index (PDI) values did not significantly change in the different FFSs analyzed compared to those of the respective controls.

### 3.4. Hemp Protein-Based Films

Preliminary experiments carried out by using NC or LF as additives in HP-based FFSs gave rise to manipulable films, whereas the presence of egagropili-derived fibers or holocellulose, hemicellulose and cellulose fractions in FFSs produced films difficult to handle, being fragile or sticky, as well as not homogeneous in their matrix. However, being biodegradable, non-toxic and possessing emulsifying properties, the extracted hemicellulose might find applications in different area of interest, such as papermaking and cosmetics industries.

Therefore, the HP-based composite films under investigation were divided into two groups based on their grafting with only two egagropili-derived additives: NC and LF. It is worthy to note that, in the presence of NC, the optimal pH value of the FFS to give rise to HP films with the best performance was pH 9, whereas the optimal pH was 12 when the reinforcement agent tested was LF.

### 3.5. Film Mechanical Properties

**Figure 4:**
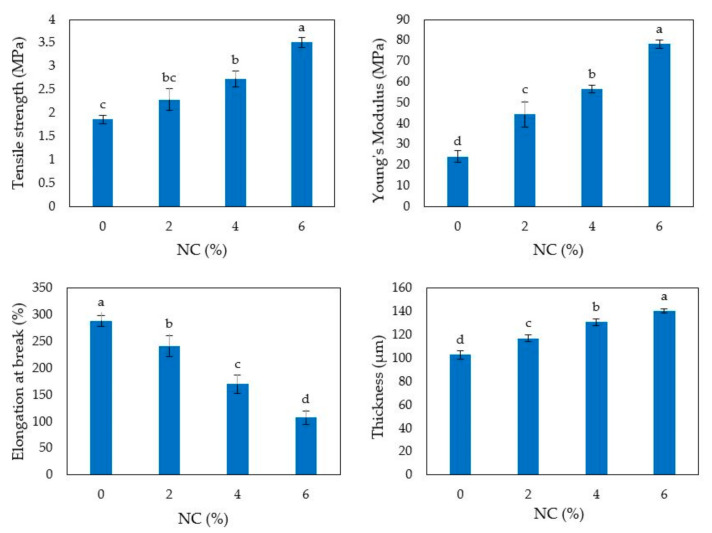
Tensile strength, elongation at break, Young’s module and thickness of hemp protein-based films derived from film forming solutions prepared at pH 9 and containing increasing amounts of egagropili nanocrystalline cellulose (NC). Different small letters a–d indicate significant differences among the values reported in each column (*p* < 0.05). Further experimental details are given in text.

The HP-based films prepared in the presence of either NC or LF were peeled intact from the casting surface and characterized for their mechanical properties. Figure 4 shows that the thickness of the nanocomposite films significantly increased by increasing the amount of NC incorporated into the film matrix.

**Figure 5:**
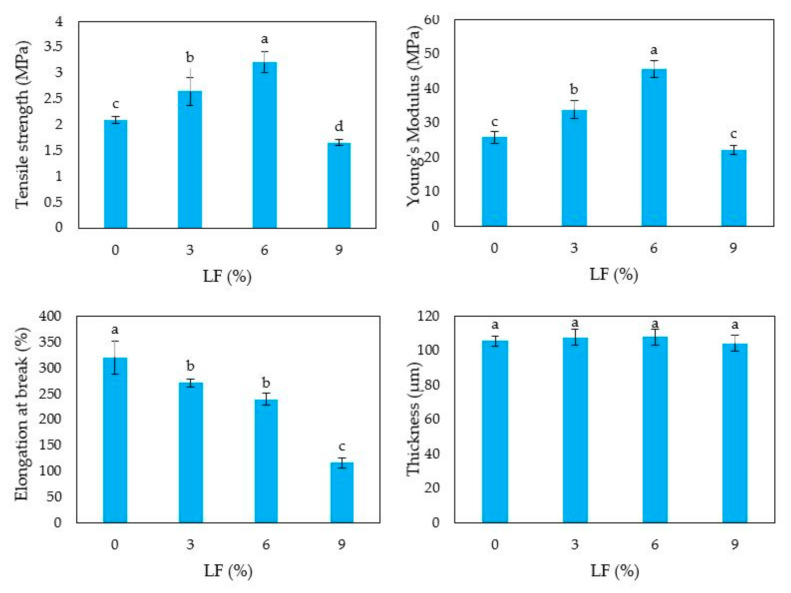
Tensile strength (TS), elongation at break (EB), Young’s module (YM) and thickness of hemp protein-based films derived from film forming solutions (FFSs) prepared at pH 12 and containing increasing concentrations of egagropili lignin fraction (LF). Different small letters a–d indicate significant differences among the values reported in each column (*p* < 0.05). Further experimental details are given in text.

Conversely, no significant effect on the film thickness was observed in the films prepared in the presence of different amounts of LF (Figure 5). Moreover, Figure 4 and Figure 5 clearly show that film TS, EB and YM were significantly affected by the addition of either NC or LF to the FFSs. In fact, both kinds of biocomposite films exhibited higher TS and YM values compared to the respective control films, whereas their EB significantly decreased as a function of NC or LF amounts occurring in the FFS. These results indicate that both NC and LF contributed to reinforce HP-based films making them more resistant but still quite flexible to be potentially applied to a variety of packaging systems. However, it is worthy to note that the addition of the highest lignin amount tested to the FFS caused a decrease of both film resistance and extensibility (Figure 5), likely due to an excessive protein aggregation.

These findings are in agreement with those previously reported by Zadeh et al. [35], who investigated the use of commercial lignin to improve the physical properties of films manufactured with transglutaminase-modified soy protein isolate, having observed an improvement in film TS due to intermolecular lignin/soy protein interactions. In addition, Alashwal et al. [36] recently reported the development of keratin-based bioplastics with improved morphology and properties when the protein was blended with nanocrystalline cellulose, envisaging their potential use in biomedical applications and manufacturing of food containers.

### 3.6. Film Moisture Content, Solubility, Swelling Ratio and Permeability

**Figure 6:**
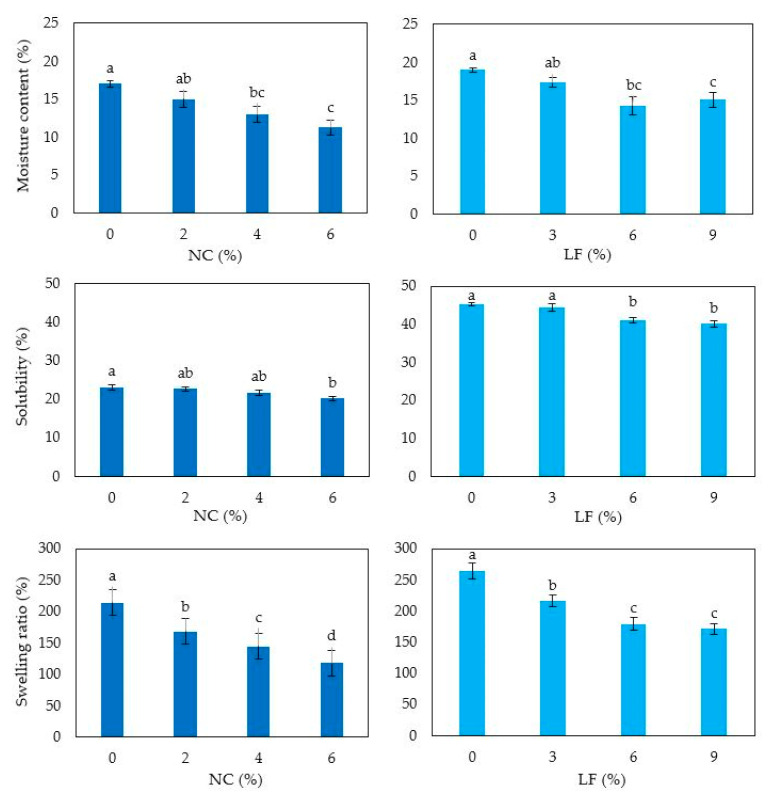
Moisture content, solubility and swelling ratio of hemp protein-based films prepared with film forming solutions containing increasing amounts of either nanocrystalline cellulose (NC) or lignin fraction (LF) and prepared at pH 9 and 12, respectively. Different small letters a–d indicate significant differences among the values reported in each column (*p* < 0.05). Further experimental details are given in text.

As water sensitivity is one of the main drawbacks of hydrocolloid bioplastics, the effect of loading NC and LF on film resistance to moisture was investigated by measuring film moisture content, water solubility as well as swelling ratio. As matter of fact, the addition of fillers generally aims not only to enhance film mechanical properties but also to confer them stability for various applications that often require film exposure to a high moisture environment. Thus, Figure 6 shows that the observed mechanical reinforcement due to the presence of either NC or LF in the HP film matrix led also to a decrease of the film moisture content, probably due to a weakened water trapping ability of the biocomposite films as a result of the denser structure of their matrix [37]. 

In addition, Figure 6 shows that also the swelling ability and solubility of the films significantly decreased, confirming that both egagropili-derived additives were able to improve film water resistance ability, probably through the formation of hydrogen bonds between the blended polymers which enhanced the cohesiveness of the matrix [38]. It is worthy to note that NC was previously exploited for improving also the moisture sensitivity of polysaccharide-based biocomposites. In this respect, Agustin et al. [39] reported that the hydrogen-bond network, formed into a starch-based matrix containing cellulose nanocrystals obtained from rice straw, prevented the formation of voids where water molecules could pass through, thus providing an increase in the resistance to the water uptake by the tested material.

**Figure 7:**
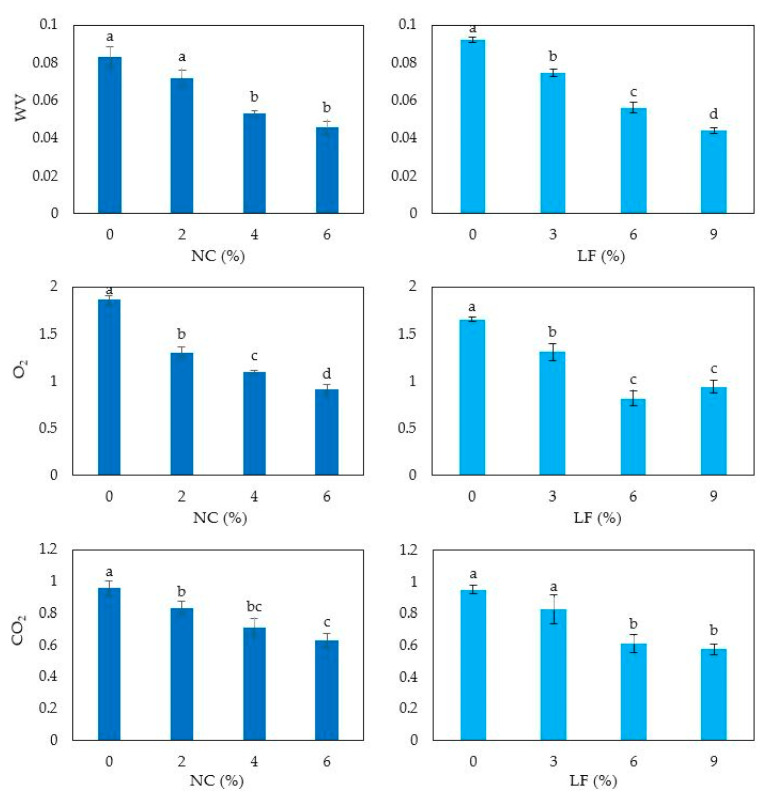
Gas and water vapor permeability of hemp protein-based films prepared with film forming solutions containing increasing amounts of either nanocrystalline cellulose (NC) or lignin fraction (LF) and prepared at pH 9 and 12, respectively. Different small letters a–d indicate significant differences among the values reported in each column (*p* < 0.05). Further experimental details are given in text.

Finally, reduced WV and gas permeability was detected in the films by increasing the concentration of both NC and LF into the FFSs (Figure 7), indicating that the observed reinforcement of the film protein matrix had a significant impact also on the barrier properties of the prepared biocomposite materials.

### 3.7. Film Morphological Properties

**Figure 8:**
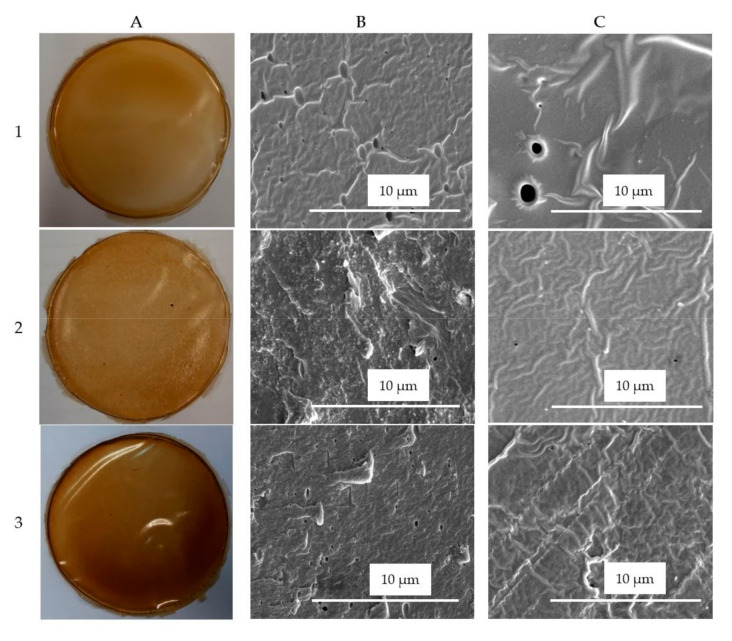
Images of hemp protein films (**A**), and of their SEM cross-sections (**B**, magnification 8000×) and surfaces (**C**, magnification 8000×), containing 50% glycerol and prepared in the absence (1) and presence of either 6% egagropili nanocristalline cellulose (2) or lignin fraction (3). Further experimental details are given in the text.

SEM analysis was carried out to examine the surface and cross-section microstructure of hemp protein-based bioplastics prepared in the absence or presence of 6% of either NC or LF. Control film, prepared in the absence of egagropili-derived fractions, was found to contain holes that interrupted the continuity of the matrix network, as evidenced in panels 1B and 1C of Figure 8 reporting the images of its cross-section and surface, respectively. Conversely, HP films containing NC (panels 2B and 2C) or LF (panels 3B and 3C) exhibited a more homogenous and uniform aspect. Hence, these results indicate a high compatibility between the film protein matrix and the reinforcement agents isolated from egagropili, consistent with the improved performance exhibited by both grafted films. The present data are in agreement with those reported by Wang et al. [40] who manufactured biocomposites by combining proteins extracted from buckwheat distiller’s dried grains with bacterial cellulose that resulted perfectly dispersed in the film matrix and endowed enhanced technological features to the obtained material.

## 4. Conclusions

Nanocrystalline cellulose and a lignin enriched fraction were isolated in good yield from egagropili, better known as sea balls, and used as reinforcement additives of a protein-based films. To this aim, new bioplastics composed of proteins extracted from hemp oilseed cakes were prepared. Film microstructure analysis indicated a good interaction of the two additives tested, as both were properly dispersed in the protein film matrix. The improved mechanical and barrier properties, as well as the weakened water trapping ability, exhibited by the grafted biocomposites suggest a potential use of egagropili, presently considered only an undesirable waste to be disposed, as a valuable renewable source for obtaining novel biodegradable materials alternative to the conventional plastics. The possible synergistic effects of nanocrystalline cellulose and lignin enriched fraction, as well as an improvement of their yield in the extraction process, remain intriguing speculations which would deserve to be further investigated.

## Figures and Tables

**Table 1:** Mean particle size, zeta potential and polydispersity index (PDI) of different hemp protein film forming solutions (FFSs) containing increasing amounts of either nanocrystalline cellulose (NC) or lignin fraction (LF) and prepared at pH 9 and 12, respectively *.

FFS Additive	Mean Particle Size (d.nm)	Zeta-Potential (mV)	PDI (%)
**None, pH 9**	374.50 ± 14.50 ^a^	−22.30 ± 1.55 ^a^	0.58 ± 0.06 ^a^
**+2% NC**	397.40 ± 10.40 ^a,b^	−21.50 ± 1.68 ^a^	0.56 ± 0.03 ^a^
**+4% NC**	466.50 ± 14.90 ^b,c^	−20.20 ± 1.30 ^a^	0.58 ± 0.03 ^a^
**+6% NC**	552.20 ± 37.54 ^c^	−21.80 ± 1.15 ^a^	0.61 ± 0.02 ^a^
**None, pH 12**	324.30 ± 8.83 ^a^	−28.50 ± 1.15 ^b^	0.54 ± 0.16 ^a^
**+3% LF**	389.80 ± 10.69 ^b^	−27.60 ± 1.06 ^b^	0.61 ± 0.31 ^a^
**+6% LF**	394.90 ± 14.35 ^b^	−27.10 ± 2.38 ^b^	0.65 ± 0.25 ^a^
**+9% LF**	312.70 ± 17.18 ^a^	−28.90 ± 1.76 ^b^	0.64 ± 0.29 ^a^

* Different small letters (a–c) indicate significant differences among the values reported in each column (*p* < 0.05). Further experimental details are given in text.
